# Atrazine removal from aqueous solutions using submerged biological aerated filter

**DOI:** 10.1186/2052-336X-11-6

**Published:** 2013-06-12

**Authors:** Mohammad Ali Baghapour, Simin Nasseri, Zahra Derakhshan

**Affiliations:** 1 Department of Environmental Health Engineering, School of Health and Nutrition, Shiraz University of Medical Sciences, Shiraz, Iran; 2 Center for Water Quality Research, Institute for Environmental Research, Tehran University of Medical Sciences, Tehran, Iran; 3 Department of Environmental Health Engineering, School of Public Health, Tehran University of Medical Sciences, Tehran, Iran

**Keywords:** Atrazine, Herbicide, Biodegradation, Submerge aerated filter, Biological filter, Aquatic environment

## Abstract

Atrazine is widely used in the agriculture as an herbicide. Due to its high mobility, Atrazine leaks into the groundwaters, surface waters, and drinking water wells. Many physical and chemical methods have been suggested for removing Atrazine from aquatic environments. However, these methods are very costly, have many performance problems, produce a lot of toxic intermediates which are very harmful and dangerous, and cannot completely mineralize Atrazine. In this study, biodegradation of Atrazine by microbial consortium was evaluated in the aquatic environment. In order to assess the Atrazine removal from the aquatic environment, submerged biological aerated filter (SBAF) was fed with synthetic wastewater based on sucrose and Atrazine at different hydraulic retention times (HRTs). The maximum efficiencies for Atrazine and Soluble Chemical Oxygen Demand (SCOD) removal were 97.9% and 98.9%, respectively. The study findings showed that Stover-Kincannon model had very good fitness (R^2^ > 99%) in loading Atrazine in the biofilter and by increasing the initial concentration of Atrazine, the removal efficiency increased. Aerobic mixed biofilm culture was observed to be suitable for the treatment of Atrazine from aquatic environment. There was no significant inhibition effect on mixed aerobic microbial consortia. Atrazine degradation depended on the strength of wastewater and the amount of Atrazine in the influent.

## Introduction

**Figure 1:**
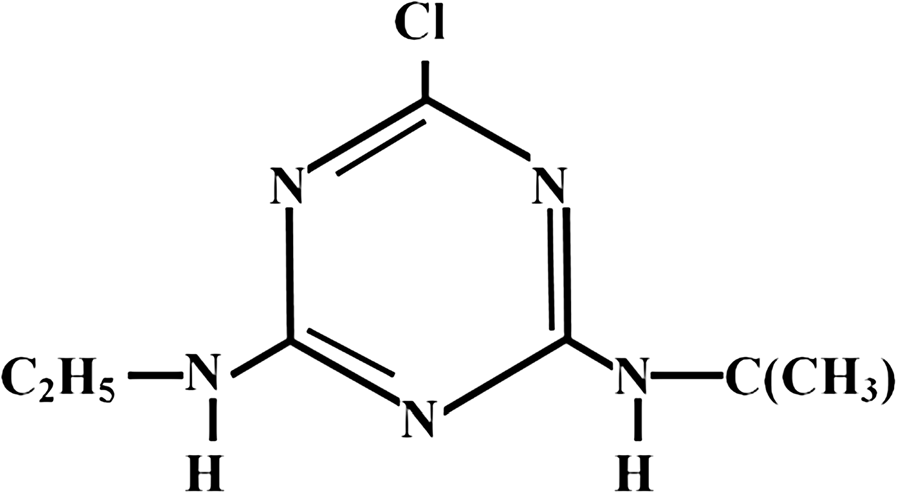
**Chemical structure of the herbicide Atrazine**[19-21]**.**

Atrazine, (2-chloro-4-ethylamino-6-isopropylamino-s-triazine), probably is the most commonly used herbicide in the agricultural activity [1-4]. Atrazine is a member of s-triazine group herbicides and is a probable human carcinogen (Group 2B) [5-7] which can cause delayed puberty, impaired development of the reproductive system and endocrine disrupting [8-10]. Atrazine is resistant in the environment and, as a result, causes serious environmental problems. Moreover, it penetrates through the surface and subsurface water bodies due to its excessive usage and high persistence and mobility [10-13]. According to the statistics of Iranian Plant Protection, 250 tons of this herbicide was used in 2008 and the average consumption was 1–5 Kg per hectare [14]. When people are exposed to Atrazine at levels above the drinking water maximum contaminant level (MCL) for relatively short periods of time, they may face congestion of heart, lungs, and kidneys, low blood pressure, muscle spasm, weight loss and damage to the adrenal gland [6,15]. Atrazine is easily absorbed through the digestive tract, skin, and lungs and chronic exposure to levels above the MCL causes heart diseases, retinal and muscle damage, weight loss, and damage to the adrenal gland [16]. Physicochemical properties and the chemical structure of Atrazine are listed in Table 1 and Figure 1, respectively.

**Table 1:** **Physicochemical properties of Atrazine**[17,18]


IUPAC^1^ Name	1-Chloro-3-ethylamino-5-isopropylamino-2,4,6-triazine
Molecular formula	C_8_H_14_ClN_5_
No. CASRN^2^	1912-24-9
Molecular weight	215.68
Physical characteristics	Solid, odorless and colorless
Density	1.23 g/cm^3^ (22°C)
Solubility in water	34.7 mg/L (22°C) and 33 mg/L (20°C)
Melting point	173 - 175°C
Boiling point	200°C
Vapor pressure	0.04 mPa (22°C)
Henry law constant	2.96 × 10^-9^ atm.m^3^/mol
Hydrolysis rate constant	2.735 × 10^-11^ cm^3^/molecule.s (25°C)
pK_a_	1.68
Log P^3^	2.71

1. International Union of Pure and Applied Chemistry.

2. Chemical Abstract Services Registry Number.

3. Octanol/water partition coefficient.

In some soils, Atrazine is stable for more than 4 years [17]. Kannan *et al*. [22] conducted a study on Lake Michigan and estimated the half-life of Atrazine in surface water to be more than 14 years. Also, Atrazine's half-life in groundwater has been reported to vary from 15 months to 20 years [23,24]. United State Environmental Protection Agency (EPA) and European Union (EU) have established the maximum amount of herbicides in drinking water in the ppb range. EU has established the permissible limit for the Atrazine as 0.1 μg/L [25-27]. However, EPA, World Health Organization (WHO.), and Institute of Standards and Industrial Research of Iran (ISIRI), have established the MCL of Atrazine in drinking water as 3, 2, and 2 μg/L, respectively [28-32].

In general, several methods are available for removing Atrazine from contaminated water and wastewater, however, these methods are very costly, have many performance problems, produce a lot of toxic intermediates, and cannot completely mineralize Atrazine. Biodegradation is an economically viable technology which may lead to complete degradation and mineralization of Atrazine and produce simple compounds, such as carbon dioxide, water, nitrogen, and organic materials. Biodegradation of Atrazine and other herbicides is the most effective option for removing these pollutants from the environment [10]. Herbicide biodegradation is a process which can occur in different environments, such as soils, sediments, surface and groundwater, and biological sludge [33,34].

Wei *et al*. [35] investigated the effects of hydraulic retention time (HRT) on the efficiency of wastewaters bearing Atrazine treatment. The study showed that when HRT reached 24 hours, Atrazine removal significantly increased. A summary of some in vitro researches performed on the microbial degradation of Atrazine is presented in Table 2.

**Table 2:** The results of some previous studies on Atrazine removal

**Operating condition/ microorganism type**	**Performance**		**Initial Conc of Atrazine (mg/L)**	**Reference**
	**Atrazine removal (%)**	**HRT**		
Aerobic/ Pure culture/ Nocardia	6 days	60	30	[36]
Aerobic/Agrobacterium radiobactor, J14a	72 hours	94	50	[37]
Aerobic/ Pure culture/ Pseudomonas	3 weeks	99	Wide range	[38]
Facultative anaerobic bacterium	1 week	47	75	[39]
Natural condition/ Natural consortia	150 days	24	11	[40]
Biostimulation with nutrients/ Pseudomonas	10 days	80	30	[41]
Phosphate/ Pseudomonas sp. ADP	4 days	75	0.01	[42]
Nocardioides and natural consortia	3 days	50	10	[43]
Anoxic/ Pure culture/ M91-3	6 days	60	22	[44]

Yang *et al*. [45] studied a simple consortium including two members of *Klebsiella sp. A1* and *Comamonas sp. A2* isolated from the sewage of a pesticide mill in China. These bacteria were able to use Atrazine alone as a source of carbon and nitrogen. The consortium showed high Atrazine-mineralizing efficiency and about 83.3% of the initial Atrazine could be degraded after 24 hours. On the contrary to many other reported microorganisms, the consortium was insensitive to some commonly used nitrogenous fertilizers. Atrazine was completely mineralized in spite of the presence of urea, (NH_4_)_2_CO_3_, and (NH_4_)_2_HPO_4_ in the medium. Wang and Xie [10] studied Atrazine removal from contaminated soil and water by *Arthrobacter sp*. and the results showed that this strain of bacteria was capable of removing Atrazine in a wide range of pH (4–11) and temperature (25- 35°C). Also, adding an external source of carbon and nitrogen increased the bacterial growth and Atrazine degradation rates.

In another research, Rezaee *et al*. [14] examined Atrazine removal by two *Pseudomonas* bacteria (*fluorescence* and *aeruginosa*) and in three concentration levels of Atrazine. The results showed that Atrazine was significantly degraded by *Pseudomonas* bacteria. During 48 hours, 48.18%, 72.6%, and 91.5% of Atrazine was degraded by *Pseudomonas fluorescence* and *Pseudomonas aeruginosa* degraded 19.08%, 33.83%, and 62.66% of Atrazine in three concentration levels of 100, 200, and 300 mg/L, respectively. They also found that increasing the Atrazine concentration led to higher degradation rates of the herbicide.

Most chemical pesticides, like Atrazine, have shown carcinogenic and mutagenic effects and removing Atrazine from the environment is a major problem. Up to now, researchers have done projects to control the transport and fate of Atrazine in the soil and aquatic environment; however, since those methods are costly, produce hazardous byproducts, and have insufficient removal efficiency, biological methods seem more economical and cost-effective. Therefore, the present study was designed with the basic objective of removing Atrazine from aqueous environment at different concentrations and HRTs by consortium of microorganisms using submerged biological aerated filter (SBAF).

## Materials and methods

### Chemicals and reagents

All used chemicals were of analytical grade and were purchased from Merck (Germany). Atrazine standard was supplied by Sigma Aldrich (USA). A stock solution of 30 mg/L Atrazine analytical grade was prepared by dissolving 3 mg solid standard of Atrazine (99.9% purity) in 100 mL methanol. Working solutions were prepared by diluting appropriate volume of the stock solution in methanol. The standard solution was stored in the freezer at −20°C. Dichloromethane was used as a solvent with analytical reagent grade (99.5% purity). Stock solutions were prepared by dissolving the required amounts of chemicals in deionized water (Milli Q). Except Atrazine, all other stock solutions were autoclaved in 120°C for 20 min and kept at 4°C. All stock solutions were kept separately and were not mixed with other stocks in order to prevent precipitation. Atrazine solution was prepared (strength 0.01 mg/L to 10.0 mg/L) by dissolving a known quantity of Atrazine in distilled water and shaking it intermittently for at least 5 days. Cartridge Atrazine solution was covered with the aluminum foil and kept at 4°C in dark in order to prevent photolytic degradation.

### Setup of biological filter

**Figure 2:**
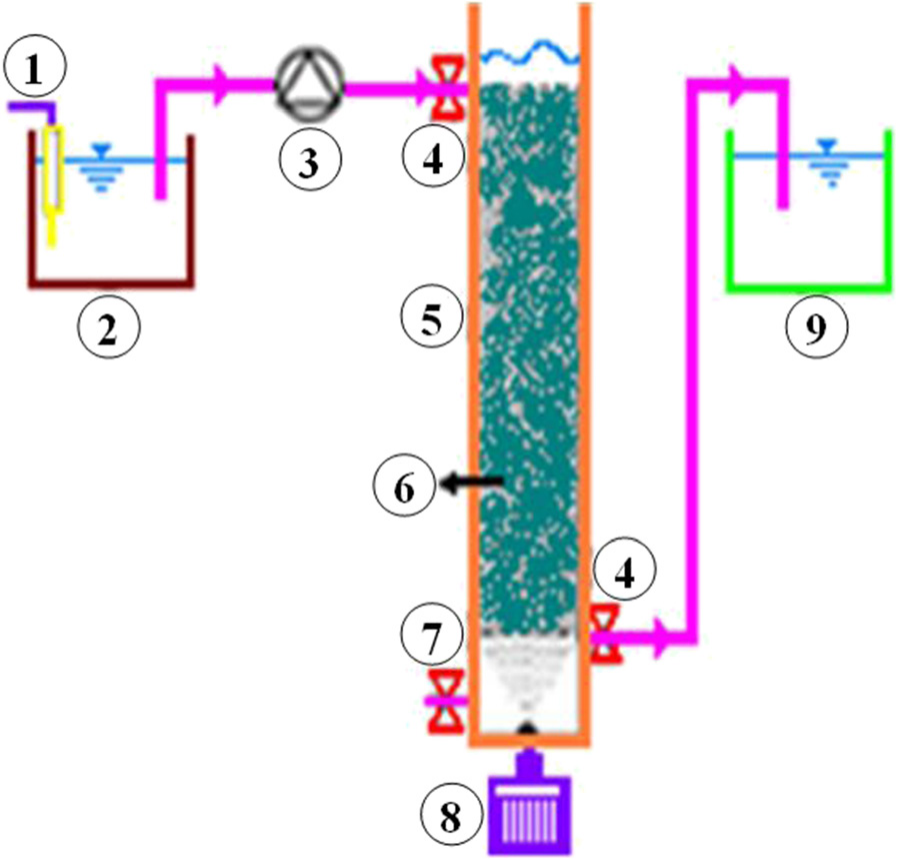
**Schematic Figure of the physical model.** 1. Temperature controller. 2. Reservoir of feed stock. 3. Peristaltic pump. 4. Sampling ports. 5. Biological aerated filter. 6. Packing media. 7. Discharge sludge port. 8. Air compressor. 9. Reservoir of outlet.

The experiments were performed in pilot scale. The physical model was setup in the School of Health, Shiraz University of Medical Sciences. A simplified flow-diagram of the pilot plant is shown in Figure 2. The model consisted of a Plexiglas column of 100 mm inside diameter as downflow submerged biological aerated filter (SBAF). The effective height of the filter and the free board were 55 cm and 5 cm, respectively. The column was filled with immobilized biofilm support of corrugated raschig rings with the same height and diameter, the rings were used as the biofilm support material because of its high porosity (up to 90%) and low price compared to other synthetic packing media. The Physical properties of the media and the physical specifications of the model are presented in Tables 3 and 4, respectively. To prevent the interference effects of light (photocatalytic) and algae growth, the column was covered by aluminum foil. Also, a control pilot was used in order to increase the accuracy of the project and eliminate the effects of the interfering factors.

**Table 3:** Physical properties of the media


Properties	Value and specification
Type media	Fixed bed (random packed)
Shape	Corrugated raschig rings
Material	HDPE^1^
Density (Kg/m^3^)	186 ± 2
Specific gravity	0.98
Porosity (%)	92
Specific area (m^2^/m^3^)	410
Thickness (micron)	350
Outside diameter (mm)	15
Inside diameter (mm)	12
Height (mm)	11-13

1. High Density Polyethylene (HDPE).

**Table 4:** Physical properties of the reactor

**Column**	**Outside diameter (mm)**	**Inside diameter (mm)**	**Height (cm)**	**V**_**t**_^**1 **^**(L)**	**V**_**e**_^**2 **^**(L)**
SBAF	160	100	60	4.7	3.9

1. Total volume.

2. Effective volume.

Aeration was done from the bottom of the BAF reactor by diffusers placed upside down. The amount of injected air was chosen in such a way that oxygen would not be a limiting factor for biological growth.

### Synthetic wastewater

The synthetic wastewater used for feeding the bioreactor was a mixture of sucrose and tap water with COD of 1000 ± 15.7 mg/L. pH fluctuations were controlled using 0.5 mol/L sodium bicarbonate. Table 5 shows the composition of wastewater used as the feed of the pilot reactor during the test period. Synthetic wastewater was injected to the top of the aerobic filter by a peristaltic pump. Based on the study by Abigail *et al.*[33] maximum removal efficiency of biodegradation Atrazine occur in 32°C. According to, in this study temperature was controlled in the reservoir at 32 ± 0.2°C by an electric heater.

**Table 5:** Chemical composition of synthetic wastewater

	**Component**	**Concentration (mg/L)**
	NaHCO_3_	20
	MgSO_4_.7H_2_O	5
	KH_2_PO_4_	5
	CaCl_2_.2H_2_O	5
Nutrients	FeSO_4_.7H_2_O	0.2
	ZnCl_2_	0.1
	CoCl_2_	0.1
	NiCl_2_	0.1
	CuSO_4_.5H_2_O	0.001
	H_3_BO_3_	0.2
	MnSO_4_	0.5
	(NH_4_)_2_HP_2_O_4_	50
	C_12_H_22_O_11_	Variable (600–900)
Atrazine	Variable (0.01, 0.1, 1 and 10)	

### Startup and system operation

The column was filled with synthetic wastewater of 10000 mg/L. In addition, seeding was provided by aerobic bacteria collected from the activated sludge system of the domestic wastewater treatment plant in Shiraz. The air compressor was then turned on and the reactors started to work in a batch condition. In aerobic conditions, the mixed bacteria are stimulated by adding oxygen to grow and start the production of enzymes which can oxidize or degrade the target pollutant. The sludge was fed with wastewater for a month to make the system acclimatized with the changed environment and was used for the further experiments. During this period, very low concentrations of Atrazine were added for further acclimatization of the microorganisms with the operational conditions.

The bacterial adaptation stage lasted about 25 days. During this time, the wastewater inside the reactors was changed four times and pH, DO, and temperature were measured as 7.5 ± 0.2, 4.8 mg/L, and 32 ± 0.2°C, respectively. Reduction of Soluble Chemical Oxygen Demand (SCOD) was also measured daily. The results of the measurements will be presented in the corresponding section. To ensure the microbial activity in this stage, surface cultivation of mixed liquor suspended solids (MLSS) in the bioreactor was frequently done in a mineral salts medium (MSM) solution containing Atrazine. The MSM preparation method was performed based on the study by Rezaee *et al*. [14].

### Experiments

After microbial adaptation completed, the continuous feeding was started. In order to assess the effect of HRT on the efficiency of the filter, wastewater with strength of 1000 mg/L was injected to the aerobic reactor by a peristaltic pump with different Atrazine concentrations (Since the range of Atrazine concentrations is highly varied in the ecosystem and depends on different factors, four logarithmic levels of Atrazine concentrations; i.e., 0.01, 0.1, 1, and 10 mg/L, were selected in this study.) and various discharges corresponding to different HRTs and different volumetric organic loads (VOLs) in the filter. The operational scheme of the system for 12 phases (Runs) is presented in Table 6.

**Table 6:** **The operational scheme of the Runs (at 32**°C**)**

**Run**	**HRT (hrs)**	**Initial Conc of Atrazine (mg/L)**	**Initial Conc of SCOD (mg/L)**	**Initial Conc of BOD**_**5 **_**(mg/L)**	**DO (mg/L)**	**pH**
1	24	0.01	992 ± 19.70	398.56	4.6 ± 0.34	7.11
2	24	0.1	996 ± 12.71	342.37	4.5 ± 0.44	7.07
3	24	1	994 ± 12.30	305.61	4.5 ± 0.40	7.08
4	24	10	995 ± 12.61	235.91	4.6 ± 0.39	7.10
5	12	0.01	998 ± 10.45	448.102	4.5 ± 0.37	7.08
6	12	0.1	998 ± 15.05	232.31	4.7 ± 0.36	7.00
7	12	1	1005 ± 5.62	299.71	4.8 ± 0.38	7.04
8	12	10	998 ± 8.14	237.85	4.7 ± 0.43	7.09
9	6	0.01	1010 ± 14.31	422.933	4.6 ± 0.47	7.01
10	6	0.1	1004 ± 14.19	358.76	4.6 ± 0.40	7.19
11	6	1	1001 ± 9.35	288.22	4.6 ± 0.41	6.98
12	6	10	991 ± 8.66	210.48	4.7 ± 0.40	7.14

Sampling was regularly carried out with 2 times repetitions and when the column reached a steady state (When difference between the measured values in consecutive measurements is less than the amount of before time, it is beginning of a steady state then with sequential measurements, extracted the mean and standard deviation of different parameters. Steady state condition for different parameters will occur almost simultaneously) regarding Atrazine residual and soluble COD, the efficiency of Atrazine and SCOD removal was determine.

The parameters measured in this research were Atrazine residual concentration, SCOD, BOD_5_, pH, dissolved oxygen (DO), and temperature. The first two parameters and the filter efficiency in Atrazine and substrate removal could be obtained in each run. In addition, at a specified HRT, pH, DO, and temperature were measured every day. To obtain rates of BOD_5_/SCOD, BOD_5_ measurements were carried out at each run. These parameters were included in the list of measurements just to be sure about proper operation of the system and stability of the reactors. Unless otherwise specified, the analyses of various parameters were done as the procedures suggested in standard methods for the examination of water and wastewater [46].

### Atrazine extraction and determination

Atrazine was extracted from wastewater by liquid–liquid extraction method suggested by Ghosh and Philip [47]. In addition, Dichloromethane (sp. gr.1.32 with Atrazine solubility of 28 g/L at 25°C) was used as the extractant. The extraction efficiency by this method was 92 ± 0.88%. Atrazine was measured by High Performance Liquid Chromatograph (HPLC) (Model: UV-2487, Water, USA) using UV/VIS detector at a wavelength of 220 nm and using Dionex Summit P580, HPLC pump. Analysis was carried out as the method reported by Yang *et al*. [45]. The analytes were filtered through a 0.22 μm nylon syringe filter. Concentration of Atrazine was determined with a reversed phase C_18_ column, 0.5 μm, 4.6 × 250 mm (Spherisorb®, Water, USA). The injection volume was 20 μL, the column working at room temperature, the mobile phase was an 80–20% methanol gradient with water, the flow rate was 0.5 mL/min, and peak retention time was 12 min. Before each run, the instruments were standardized with anticipated Atrazine concentration range. For standardization of the instrument, six standards of Atrazine were prepared in advance and stored in an amber bottle in the refrigerator at 4°C until use. The standards were prepared by serial dilutions. To check the build-up of Atrazine in biofilm, the method suggested by Ghosh and Philip [47] was utilized.

### Modeling

In almost all references, including Baghapour *et al*. [48], it is confirmed that the criterion for submerged filters design is the volumetric organic load (VOL) and the rate of substrate removal is obtained from hyperbolic relations, such as Stover-Kincannon function (equation 1). The Stover–Kincannon model was first proposed for a rotary biological contactor by Kincannon and Stover [49]. The original model assumed that the suspended biomass was negligible in comparison to the attached biomass to the media [13,50].

(1) $$r_{\mathrm{ATZ}}=r_{\max}\frac{B_{\mathrm{ATZ}}}{k+B_{\mathrm{ATZ}}}$$

Where r_ATZ_ is the volumetric Atrazine removal, r_max_ is the maximum rate of volumetric Atrazine removal, B_ATZ_ is the Atrazine load per unit volume of the filter, and k is the constant of half velocity. All the parameters are in Kg_Atrazine_/m^3^d.

The values of B_ATZ_ and r_ATZ_ could be obtained from the following equations:

(2) $$B_{\mathrm{ATZ}}=\frac{Q}{V}C_{i}$$

(3) $$r_{\mathrm{ATZ}}=\frac{Q}{V}\left(C_{i}-C_{e}\right)$$

C_i_ is Atrazine concentrations in the influent (Kg_Atrazine_/m^3^)

C_e_ is Atrazine concentrations in the effluent (Kg_Atrazine_/m^3^)

Using equations 2 and 3 and Tables 6 and 7, values of B_ATZ_ and r_ATZ_ could be computed for various situations. The main values are presented in Table 8. The values of k and r_max_ were obtained by using the software Curve Expert and are presented in Table 9

**Table 7:** **Effluent concentration of Atrazine and SCOD and their removal efficiencies from the bioreactor in steady state at 32**°C

**Run**	**Output Conc of Atrazine (mg/L)**	**Output Conc of SCOD (mg/L)**	**BOD**_**5 **_**/ SCOD**	**Removal Efficiency (%)**	
				**Atrazine**	**SCOD**
1	0.0028 ± 3 × 10^-5^	17.15 ± 0.707	0.8	71.8	98.9
2	0.0204 ± 1 × 10^-4^	78.52 ± 1.050	0.79	79.6	97.8
3	0.123 ± 1 × 10^-3^	243.28 ± 1.773	0.78	87.7	92.2
4	0.2012 ± 2 × 10^-2^	324.95 ± 0.889	0.77	97.9	98.3
5	0.0036 ± 4 × 10^-5^	32.51 ± 1.143	0.8	63.4	96.7
6	0.0240 ± 3 × 10^-4^	190.53 ± 3.503	0.78	75.8	93.3
7	0.1571 ± 1 × 10^-3^	288.23 ± 2.189	0.77	84.3	90.1
8	0.3899 ± 2 × 10^-2^	353.78 ± 1.169	0.76	96.1	94.9
9	0.0039 ± 1 × 10^-5^	89.38 ± 3.103	0.78	61.6	92.3
10	0.0300 ± 9 × 10^-5^	216.60 ± 2.237	0.73	70.1	89.2
11	0.209 ± 1 × 10^-3^	309.42 ± 1.522	0.67	79.1	87.5
12	0.6101 ± 2 × 10^-2^	368.99 ± 1.655	0.65	93.9	91.2

The number of repetitions in each run after steady state = 3.

**Table 8:** **Volumetric load and removal of Atrazine and SCOD from the bioreactor at 32**°C

**Run**	**B**_**ATZ **_**(Kg**_**Atrazine**_**/m**^**3**^**d)**	**r**_**ATZ **_**(Kg**_**Atrazine**_**/m**^**3**^**d)**	**B**_**SCOD **_**(Kg**_**SCOD**_**/m**^**3**^**d)**	**r**_**SCOD **_**(Kg**_**SCOD**_**/m**^**3**^**d)**
1	9.2 × 10^-6^	6.60 × 10^-6^	0.920	0.910
2	9.2 × 10^-5^	7.32 × 10^-5^	0.920	0.899
3	9.2 × 10^-4^	8.07 × 10^-4^	0.920	0.848
4	9.2 × 10^-3^	9.007 × 10^-3^	0.920	0.904
5	1.84 × 10^-5^	1.17 × 10^-5^	1.840	1.779
6	1.84 × 10^-4^	1.4 × 10^-4^	1.840	1.716
7	1.84 × 10^-3^	1.551 × 10^-3^	1.840	1.657
8	1.84 × 10^-2^	1.7682 × 10^-2^	1.840	1.746
9	3.68 × 10^-5^	2.27 × 10^-5^	3.680	3.396
10	3.68 × 10^-4^	2.58 × 10^-4^	3.680	3.282
11	3.68 × 10^-3^	2.911 × 10^-3^	3.680	3.22
12	3.68 × 10^-2^	3.455 × 10^-2^	3.680	3.356

**Table 9:** **k and r**_**max **_**coefficients of the bioreactor at 32**°C **for Stover–Kincannon model**

	**Atrazine**	**SCOD**
r_max,_ (Kg/m^3^d)	1.1781	39.0296
k, (Kg/m^3^d)	1.2211	39.6738
R^2^	0.999	0.999

## Results

**Figure 3:**
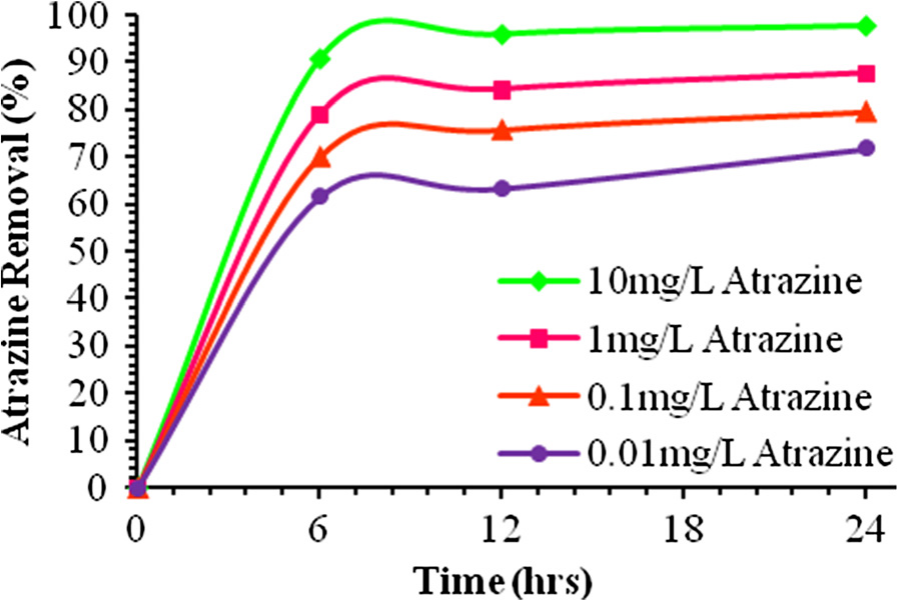
Trend of removing Atrazine in bioreactor at 32°C.

**Figure 4:**
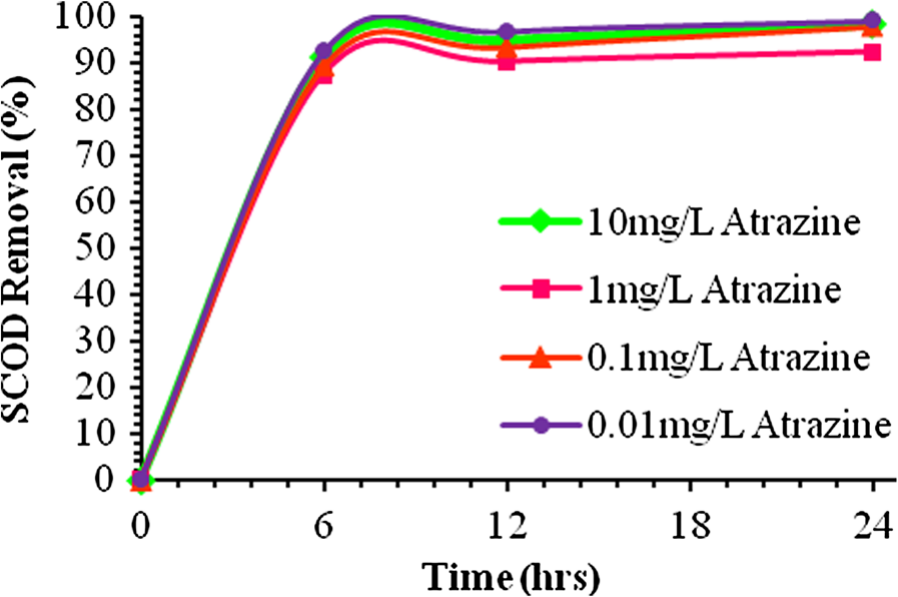
Trend of SCOD removal in bioreactor at 32°C.

During the system operation period, the HRT was reduced from 24 to 12 hours and then to 6 hours. According to the HRTs, the flow rate in the reactor was set at 0.1504, 0.3009, and 0.6018 L/hr, respectively. The most important parameters monitored in the experiments were Atrazine residual and SCOD and the means of the measured data are reported in this paper (Table 7). COD of the inflow wastewater in all situations was 1000 ± 15.75 mg/L. Trend of Atrazine and SCOD removal shown in Figures 3 and 4.

**Figure 5:**
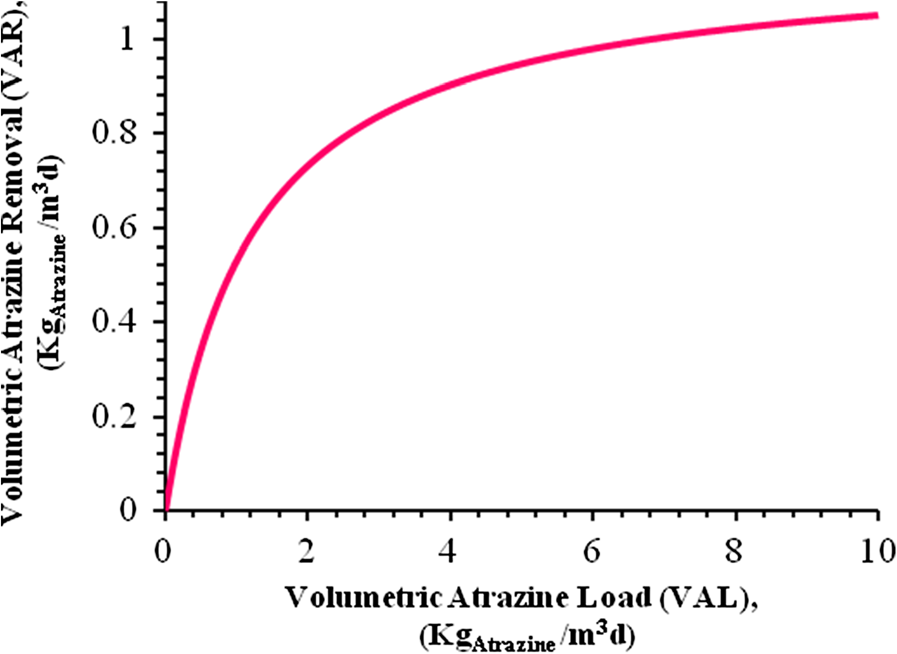
**Atrazine loading of the bioreactor in the range of 0 to 10Kg**_**Atrazine**_**/m**^**3**^**d at 32°C.**

**Figure 6:**
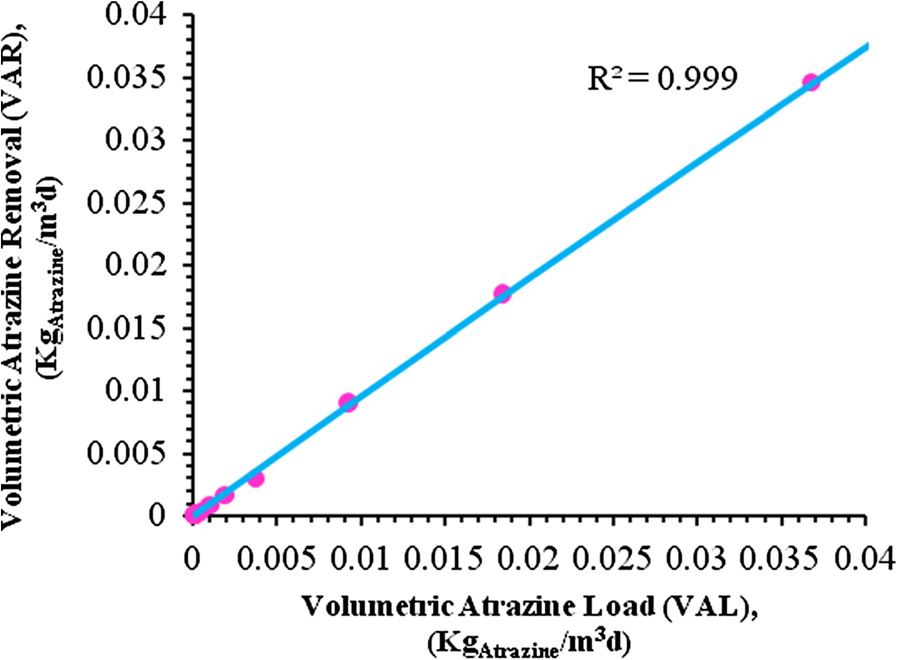
**Atrazine loading of the bioreactor in the range of 0 to 0.04Kg**_**Atrazine**_**/m**^**3**^**d at 32°C.**

**Figure 7:**
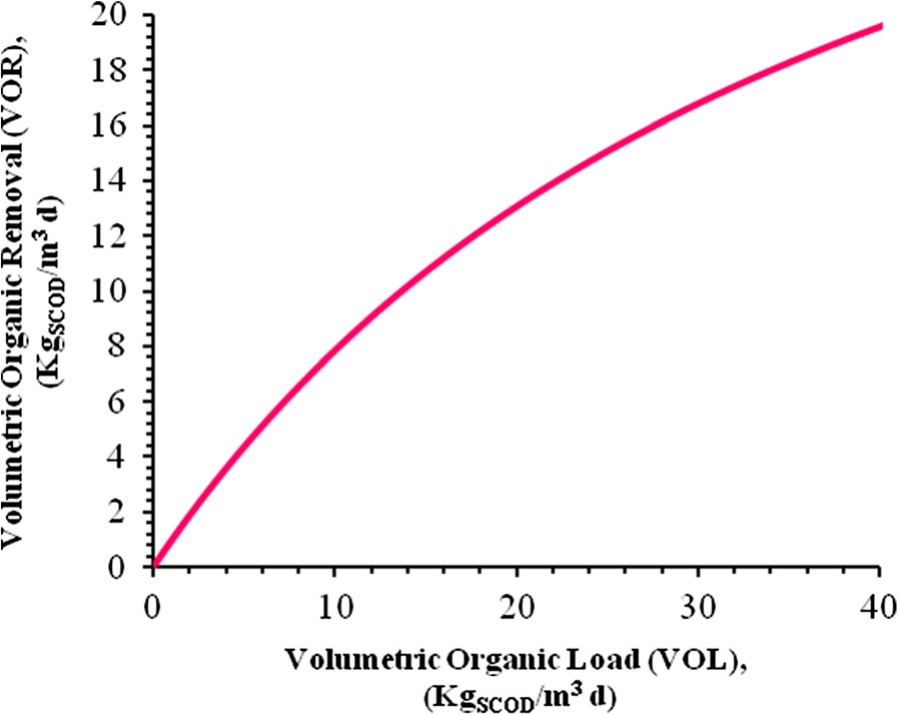
**Organic loading of the bioreactor in the range of 0 to 40Kg**_**SCOD**_**/m**^**3**^**d at 32°C.**

**Figure 8:**
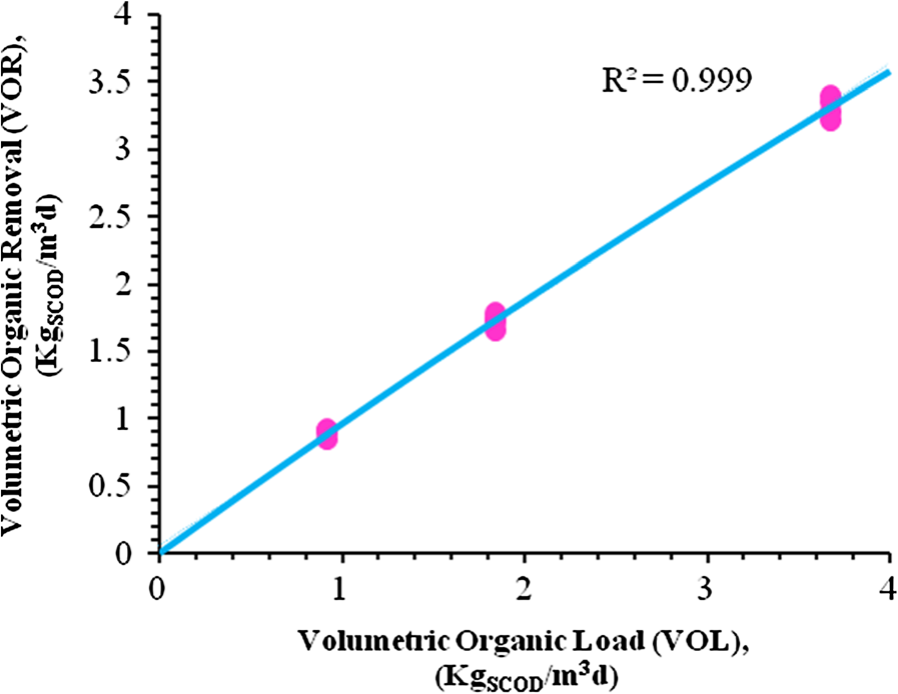
**Organic loading of the bioreactor in the range of 0 to 4Kg**_**SCOD**_**/m**^**3**^**d at 32°C.**

By substitution of the values Table 9 into Eq. 1, results presented in Figures 5, 6, 7 and 8 are obtained and submerged filters could be designed using these diagrams.

At the initial Atrazine concentration of 0.01, 0.1, 1 and 10 mg/L, Atrazine removal efficiency after 6 hrs, were 61.6%, 70.1%, 79.1% and 93.9% respectively. After 12 hrs, however, Atrazine removal efficiency in the reactor reached 63.4%, 75.8%, 84.3%, and 96.1% respectively. Finally, after 24 hrs, Atrazine removal in reactor was 71.8%, 79.6%, 87.7%, and 97.9% at the initial Atrazine concentration of 0.01, 0.1, 1 and 10 mg/L, respectively (Table 7). In steady state conditions at HRT of 6 hrs and the initial Atrazine concentrations of 0.01, 0.1, 1, and 10 mg/L, the average SCOD removal was 92.3%, 89.2%, 87.5%, and 76.8%, respectively. Besides, at HRT of 12 hrs and the initial Atrazine concentrations of 0.01, 0.1, 1, and 10 mg/L SCOD removal efficiency was 96.7%, 93.3%, 90.1%, and 94.9%, respectively. Finally, the average SCOD removal efficiency was 98.9%, 97.8%, 92.2%, and 98.3% at HRT of 24 hrs and the initial Atrazine concentrations of 0.01, 0.1, 1, and 10 mg/L, respectively. In all the cycles of the operation, SCOD removal efficiency and effluent BOD_5_/SCOD were more than 87% and 0.65, respectively.

## Discussion

Based on the results Atrazine degradation potential of the mixed aerobic consortium was evaluated under various Atrazine concentrations and HRTs and the results were presented in Tables 6 and 7. The findings of this study demonstrated that solution containing Atrazine was easily biodegraded and treated in a submerged biological aerated filter. Moreover, Atrazine removal efficiencies were above 94% where high Atrazine influent was introduced in the SBAF (runs 4, 8 and 12). The major part of the input Atrazine was consumed during these runs as indicated by low effluent Atrazine concentration (below 0.6101 ± 2 × 10^-2^ mg/L). The treatment efficiencies achieved at longer HRT (24 hrs) in the SBAF fed with low, moderate, and high Atrazine concentrations in the influent are summarized in Table 7. It is evident that in comparison with other HRTs, Atrazine and SCOD removal efficiencies were increased at long HRT due to the slight decrease in Atrazine and organic loading rates in the SBAF. However, the extent of Atrazine loading rate was not highly effective in biological Atrazine and organic removal efficiencies. Afterwards, the HRT was set to 24 hrs and the SBAF was operated at these conditions until steady state conditions were reached. The Atrazine and SCOD removal efficiencies were increased up to 72% and 92% respectively (Table 7). Therefore, it can be concluded that decreasing Atrazine as well as organic loading positively affect the SBAF performance. This can due to the increase of probability of the contaminants exposure with microbial consortium, which is consistent with the results obtained by Gosh *et al*. [47] and Rezaee *et al*. [14]. Measurement of COD is important regarding the effluent discharge standards and COD represents the treatment potential of the reactor. In this study, SBAF showed acceptable SCOD removal efficiency in all experiments. Besides, Atrazine revealed no adverse effects on SCOD removal up to the concentration of 10 mg/L. However, SCOD reduction was reduced by 2–6% when Atrazine concentration was increased to 0.1 and 1 mg/L, which is in agreement with the results of the study by Gosh *et al.*[47]. Comparison of the results of the previous studies (Table 2) and the present one shows that this system has high ability for removing Atrazine from aqueous solutions. There was no accumulation of Atrazine in the biofilm and the loss of Atrazine in the control reactor was negligible. This shows that Atrazine removal from the system was due to biodegradation. High degradation rate of Atrazine at comparatively high Atrazine concentration might be due to the effect of concentration gradient. At high concentration gradient, the pollutant has a higher chance to be exposed to and/or penetrate through the cell which is essential for biodegradation. BOD_5_ is a measure of the oxidation occurring due to microbial activity. The BOD_5_/COD ratios are the commonly used indicators of biodegradability improvement where a value of zero indicates nonbiodegradability and an increase in the ratio reflects biodegradability improvement. In this study, the SBAF was able to increase the BOD_5_/COD ratio to more than 0.65 in all the experiments. Moreover, significant changes were observed in BOD_5_/COD ratios by increasing the HRT.

Co-metabolic process is used for bioremediation of most persistence contaminants, such as Atrazine. In co-metabolic processes, by utilizing primary carbon source or nitrogen source, microbes produce enzymes or cofactor during microbial activities which are responsible for degradation of the secondary substrates (toxic compounds, Atrazine). Also, the contaminants degrade in this process in order to trace concentrations. The results obtained from SBAF showed that the co-metabolic process was quite effective in removing Atrazine from the aqueous environment. Additional nitrogen sources (ammonium phosphate) also showed no adverse effects on Atrazine degradation. Similar results were also reported by Yang *et al*. [45]. Overall, the results of the modeling showed that Stover – Kincannon model had a very good fitness (R^2^ > 99%) in loading Atrazine in this biofilter, which is in line with the findings of Cheyns *et al*. [51].

## Conclusion

The present study investigated the ability of a Submerged Biological Aerated Filter (SBAF) to remove Atrazine from aqueous environment. The SBAF was operated at 3 different aerobic retention times in order to determine the optimum retention time for the highest Atrazine and COD removal. Finally, aerobic mixed biofilm culture was observed to be suitable for the treatment of Atrazine from aqueous solutions. There was no significant inhibition effect on mixed aerobic microbial consortia. Atrazine degradation depended on the strength of wastewater and the amount of Atrazine in the influent and HRTs. Also, Stover-Kincannon model more desirably described the Atrazine degradation in aquatic environment using a SBAF.

## Competing interests

The authors declare that they have no competing interests.

## Authors’ contributions

MAB participated in the design of study, coordinated activities and revised manuscript. SN participated in the design of the study, final revised of manuscript and intellectual helping for analyzing of data. ZD performed data collection, carried out statistical and technical analysis of data, participated in design of study and drafted manuscript. All authors read and approved the final manuscript.
